# Supplementary figures and images for: A novel binary pesticidal protein from Chryseobacterium arthrosphaerae controls western corn rootworm by a different mode of action to existing commercial pesticidal proteins
Source: PLoS One. 2023 Feb 17;18(2):e0267220. doi: 10.1371/journal.pone.0267220 (PMC9937505; doi:10.1371/journal.pone.0267220)

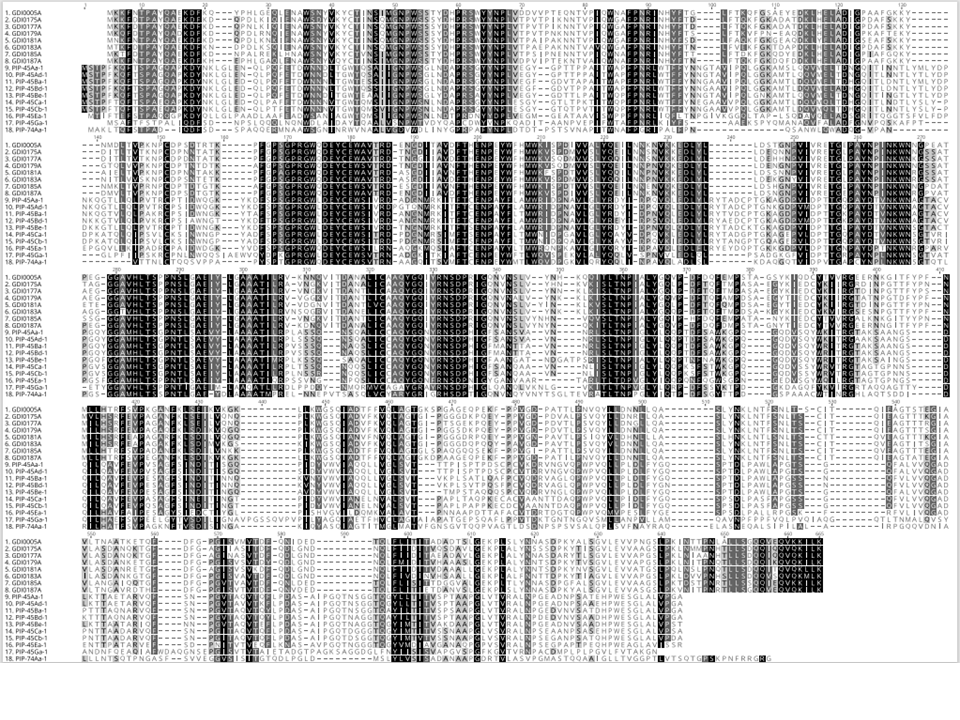

Supplement: S1 Fig — All sequence information is presented in the S1 Table. GDI proteins are from this study, PIP sequences are from the patent WO2016/114973. (TIF) [file pone.0267220.s001.tif]

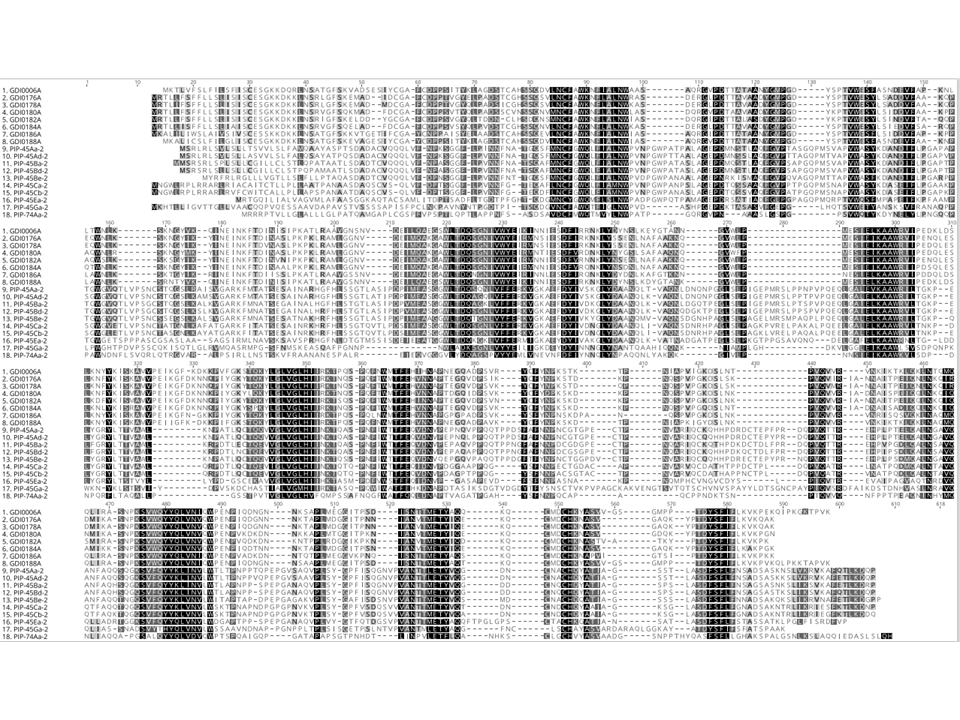

Supplement: S2 Fig — All sequence information is presented in the S1 Table. GDI proteins are from this study. PIP sequences are from patent WO2016/114973. (TIF) [file pone.0267220.s002.tif]

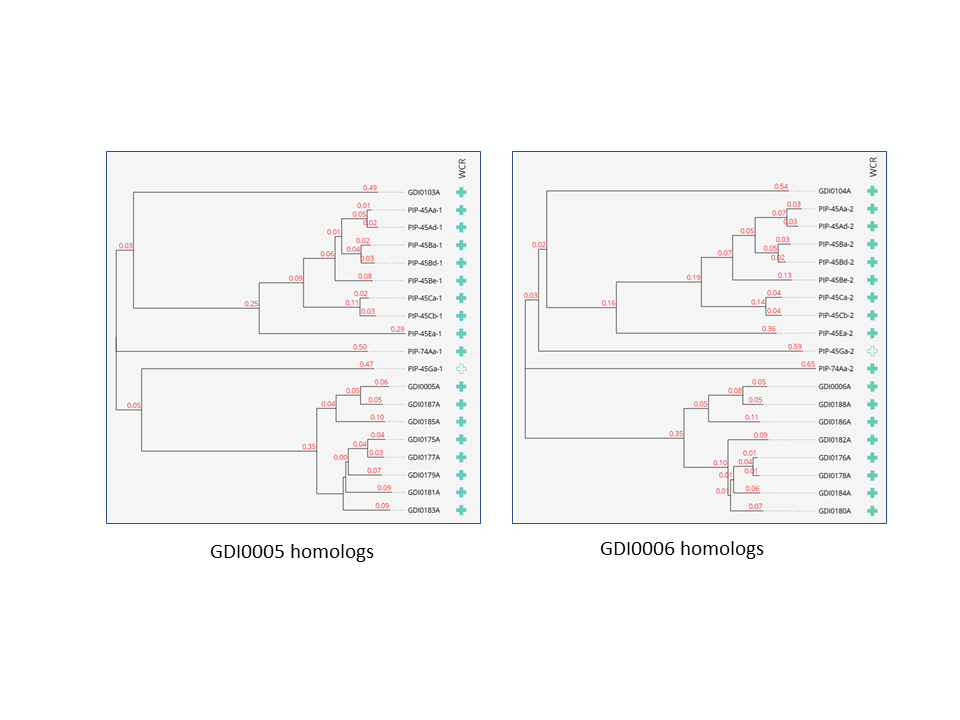

Supplement: S3 Fig — Phylogenetic tree was built with the Neighbor-joining method using Jukes-Cantor Genetic Distance model (Geneious Tree Builder from Geneious). (TIF) [file pone.0267220.s003.tif]

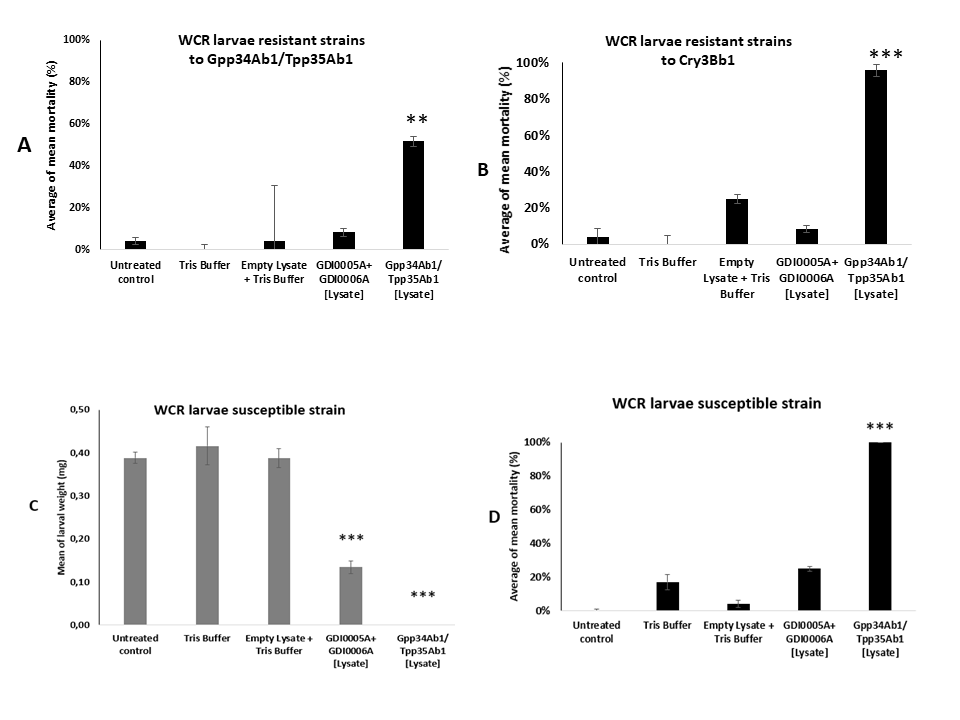

Supplement: S4 Fig — (A), (B), and (D) describe the mortality and (C) describes the larval weight 5 days after treatment in diet overlay bioassays. (TIF) [file pone.0267220.s004.tif]

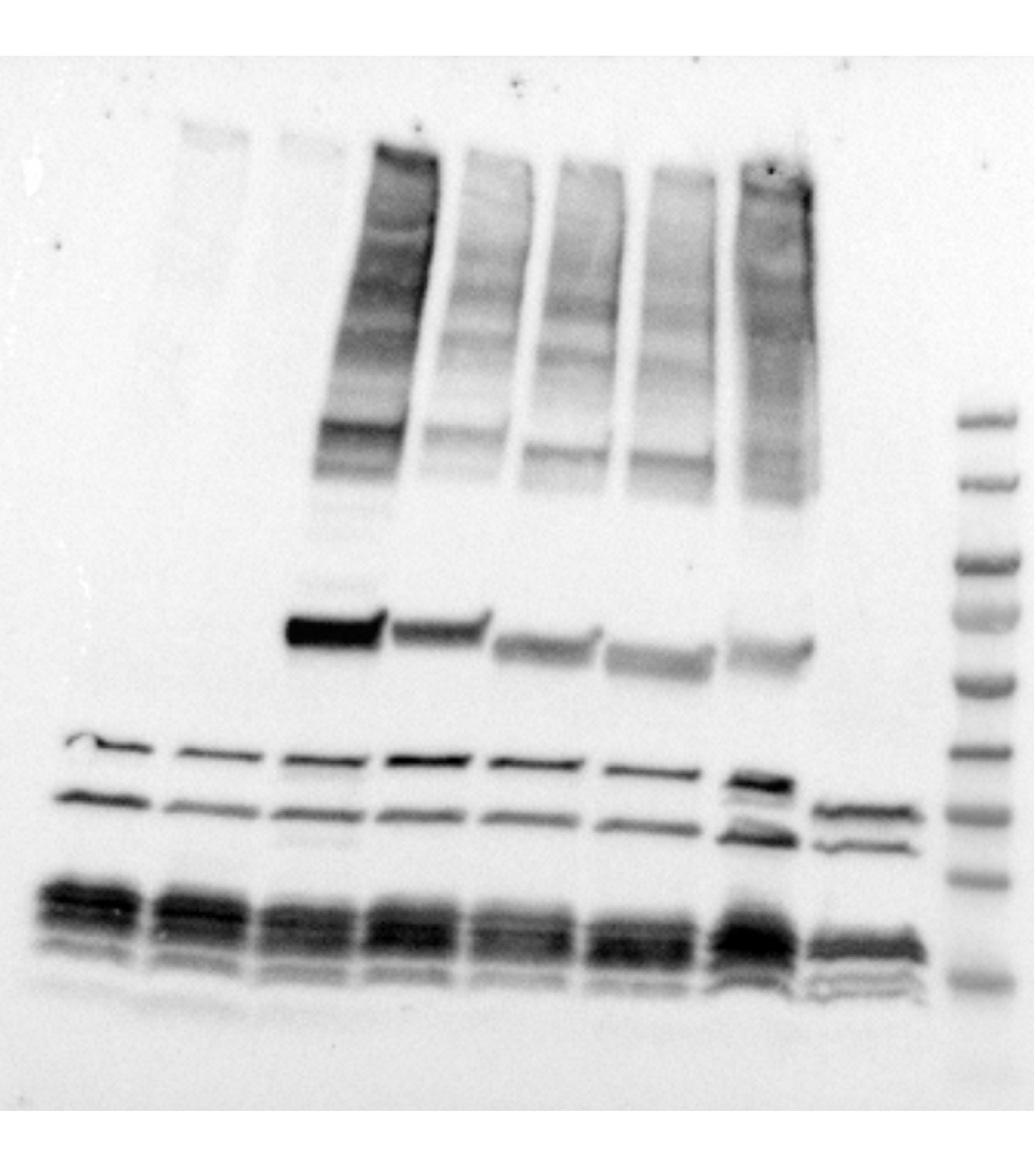

Supplement: S1 Raw image — (JPG) [file pone.0267220.s008.jpg]
